# Effect of glenoid concavity loss on shoulder stability- a case report in a professional wrestler

**DOI:** 10.1186/s12891-016-1210-9

**Published:** 2016-08-22

**Authors:** Philipp Moroder, Franziska Haniel, Michael Quirchmayr, Eva Schulz, Manfred Eppel, Nicholas Matis, Alexander Auffarth, Herbert Resch

**Affiliations:** 1 Department of Traumatology and Sports Injuries, Paracelsus Medical University, Muellner Hauptstrasse 48, 5020 Salzburg, Austria; 2 Center for Musculoskeletal Surgery, Campus Virchow, Charité -Universitaetsmedizin Berlin, Augustenburgerplatz 1, 13353 Berlin, Germany

**Keywords:** Bony Shoulder Stability Ratio, Bony Bankart repair, Shoulder instability, Glenoid defect

## Abstract

**Background:**

Current glenoid defect measurement techniques only quantify bone loss in terms of defect diameter or surface. However, the glenoid depth plays an important role in shoulder stabilization by means of concavity compression.

**Case presentation:**

We present a case of a professional wrestler who suffered from anterior shoulder instability after sustaining a bony Bankart lesion without loss of glenoid surface area but flattening of the concavity due to medialization of the fragment. The patient’s glenoid concavity was reconstructed arthroscopically by reduction and percutaneous screw fixation of the bony fragment along with a capsulo-ligamentous shift. Changes of the glenoid concavity with according alterations in the Bony Shoulder Stability Ratio (BSSR) were analyzed on pre-op, post-op, and follow-up CT scans. Postoperative CT scans revealed a deepened concavity (3.3 mm) and improved BSSR (46.1 %) compared to pre-op scans (0.7 mm; 11.3 %). Follow-up CT scans showed a slight remodeling of the glenoid concavity (3.2 mm) with steady BSSR (44.7 %).

**Conclusion:**

This case shows that the passive stabilizing effect of the glenoid can be compromised by loss of concavity despite the absence of loss of articular surface. Therefore, addressing the concavity loss and resulting reduction of the BSSR is recommended in these cases. Bony Bankart repair was successful in restoring the BSSR of the patients shoulder as determined by mathematical calculations based on CT scans.

## Background

Glenoid Defects are common injuries associated with recurrent anterior shoulder instability [1]. Typically an anterior glenoid rim defect occurs either in form of attritional bone loss or a fracture with resulting free bony fragment. Such fragments typically medialize and resorb over time [2] and eventually either disappear or remain visible in form of a small ridge slightly medial to the anterior glenoid rim [3]. The size of the osseous defect is critical for shoulder stability and has been discussed in previous reports [4–6].

**Fig. 1:**
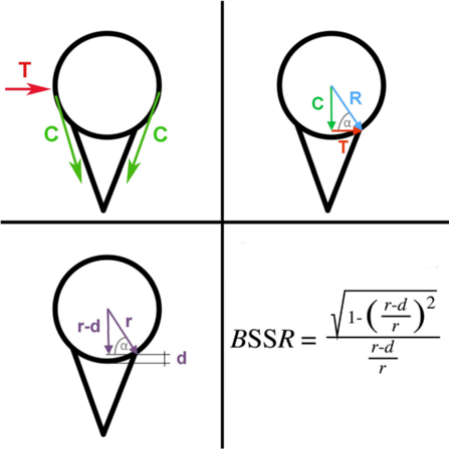
Schematic drawings of a gleno-humeral joint with illustrated translational force (T), compressive force (C), resulting joint force vector (R), joint radius (r), and concavity depth (d). The geometrical measurements and force vectors can be used to calculate the Bony Shoulder Stability Ratio (BSSR) as previously described [10]

A recent CT study showed that glenoid bone loss not only results in reduction of glenoid surface area but also in a flattening of the glenoid concavity [7]. A loss of glenoid concavity compromises the concavity compression exerted by the rotator cuff, which is the main stabilizing mechanism of the shoulder in mid-range of motion [5, 8, 9]. These changes in stability due to morphological alterations of the bony glenoid concavity can be mathematically quantified in vivo by calculating the Bony Shoulder Stability Ratio (BSSR) based on CT measurements [10] (Fig. 1).

We present the case of a professional wrestler with anterior shoulder instability without loss of glenoid surface area but loss of glenoid concavity due to medialized malunion of a bony glenoid rim fragment. The biomechanical effects of arthoscopic mobilization, reduction and percutaneous screw fixation of the bony fragment on the morphology of the glenoid concavity and the resulting BSSR are analyzed according to a previously published method [10] and the clinical outcome is reported.

## Case presentation

**Fig. 2:**
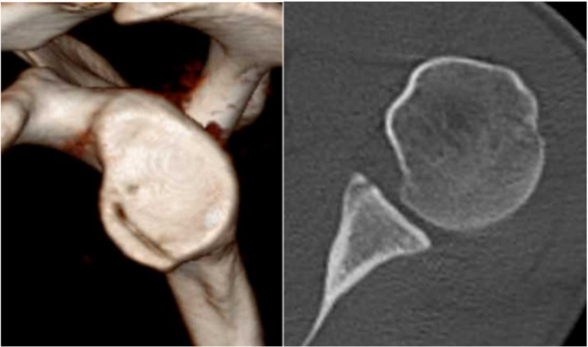
No loss of glenoid surface area or diameter can be detected on the preoperative 3DCT scans. However, a dimished glenoid concavity can be detected on the axial CT scans

A 31-year old male professional wrestler was referred to our department by his primary care physician because of recurrent uni-lateral anterior shoulder instability episodes and anterior shoulder pain preventing participation in competition and practice. The first reported instability episode was a traumaic subluxation which had occurred a year earlier during a wrestling fight. Multiple further subluxations ensued afterwards. The patient had not undergone any previous surgical interventions on his shoulder. During clinical examination the patient featured a positive apprehension- and relocation test, a negative sulcus-sign and a Beighton Score of 0. No loss of range of motion or strength was noted. CT scans with 3D reconstruction revealed no lack of glenoid surface area, however flattening of the glenoid concavity due to fracture and medialized malunion of the anterior glenoid rim was visible on axial CT scans (Fig. 2). Preoperative MRI examination showed no detachment of the labrum from the medialized anterior glenoid rim. The patient was treated with arthoscopic osteotomy, reduction and percutaneous screw fixation of the bony fragment along with arthroscopic capsulo-ligamentous shift.

### Surgical technique

**Fig. 3:**
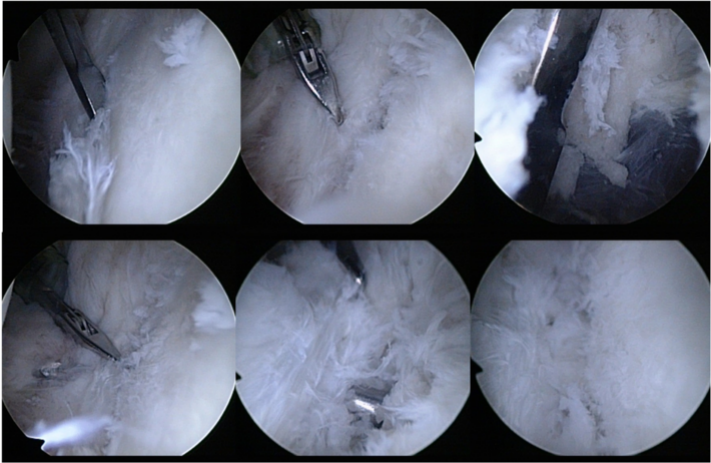
Intraoperative arthroscopic images show the impacted anterior glenoid rim with attached capsulo-labral complex which was mobilized, reduced, and stabilized by percutaneous screw insertion as described by Tauber et al. [11]

The surgical technique has previously been described [11]. The operation was performed in beach chair position and under general anaesthesia. Diagnostic arthroscopy revealed that the articular surface of the anterior glenoid rim was slightly medialized resulting in a reduced concavity depth as visible on the preoperative CT scans. The capsulo-labral complex was still attached to the medialized osteoarticular fragment without any visible Bankart lesion. Only a small and shallow Hill-Sachs lesion was detected. The fragment was mobilzed using a periostal elevator and subsequently reduced. A percutaneous fracture screw system was used to fix the fragment with a cannulated self-tapping screw inserted from antero-inferiorly into the gleno-humeral joint (Fig. 3). Finally the anteroinferior capsuloligamentous complex was moderatly tightened using two knotless suture anchors. The Patient was discharged from the hospital on the third postoperative day and had to wear a shoulder sling for 4 weeks before starting physiotherapy.

### Clinical outcome

**Fig. 4:**
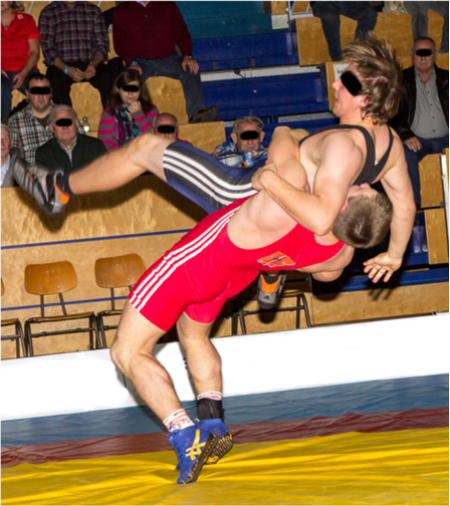
The patient competing in a wrestling competition 6 months after surgery

The post-operative clinical improvement of the patient was recorded prospectively at 3, 6, and 12 months. The Western ontario shoulder instability index (WOSI, range 2100–0 points) improved from 895 preoperatively to 355, 142 and 37 points, respectively. The Rowe Score (range 0–100 points) increased from 47 preoperatively to 87, 97, and 97 points, respectively. Similar to the Subjective Shoulder Value (SSV, range 0–100 %) which increased from 85 to 93, 96, and 99 %, respectively. The Athletic Shoulder Outcome Scoring System (ASOSS, range 0–100 points) increased from 52 to 84, 96, and 96 points, respectively. At the 6 and 12 months follow-up the patient featured a negative apprehension test and the range of motion as well as strength of the affected shoulder was fully restored. He started actively participating in wrestling competitions 5 months after surgery without any events of shoulder instability (Fig. 4).

### Radiological outcome

**Fig. 5:**
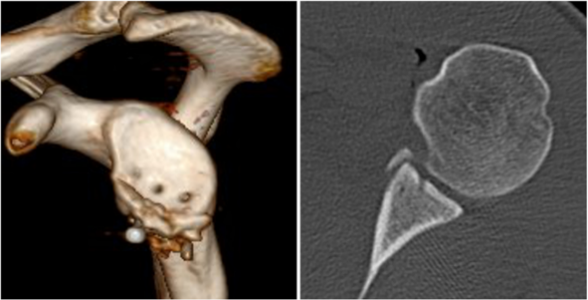
The postoperative CT scans 1 day after surgery show the reconstructed anterior glenoid rim with deepened glenoid concavity

**Fig. 6:**
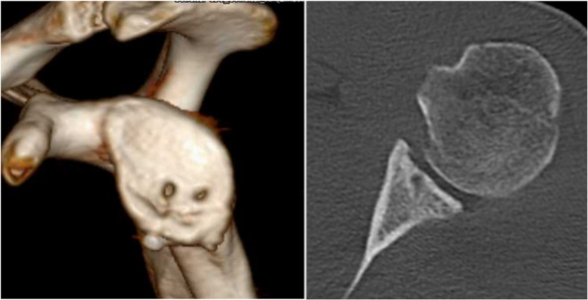
A slight remodeling process of the reconstructed glenoid concavity is visible 1 year after surgery. The screw head is prominent however not lying intaarticular, therefore resulting in no restraints for the patient

Based on the glenoid concavity depth and radius measured on a standarized axial imaging plane [7] on preoperative, postoperative, and 1-year follow-up CT scans the BSSR was calculated utilizing a previously published and validated mathematical formula [10]. The preoperative CT scans revealed a glenoid concavity depth of 0.7 mm and a BSSR of 11.3 % compared to 3.1 mm and 40.3 % measured on the unaffected contralateral side (Fig. 2). Postoperative CT scans showed a deepened concavity (3.3 mm) and improved BSSR (46.1 %) compared to the pre-op scans (Fig. 5). The follow-up CT scans showed a slight remodeling of the glenoid concavity (3.2 mm) with steady BSSR (44.7 %) (Fig. 6).

## Discussion

Biomechanical studies have revealed that glenoid bone loss in anterior shoulder instability results in a decreased stability ratio of the joint. A loss of the glenoid arc was deemed responsible for the decrease in gleno-humeral stability [5]. However, current glenoid defect measurement techniques only consider the glenoid articular surface in two dimensions and bone loss is mostly quantified either in terms of the defect diameter or surface [12–14].

In a recent study, a mathematical formula was introduced that allows for in-vivo determination of the stability ratio of the bony glenoid concavity based on CT scans [10]. In the study it was also revealed that patients with shoulder instability do not feature smaller but inherently flatter glenoids than healthy controls with resulting reduced stability ratios [10].

We presented the case of a patient with bony Bankart lesion and slightly medialized malunion of the fragment. Even though no loss of glenoid articular surface area or diameter was detected, the BSSR was significantly reduced due to the loss of glenoid concavity depth. The arthroscopic intervention including fragment mobilization, reduction, and fixation was successful in significantly increasing the BSSR to 46 % which is four times higher than the preoperative value and slightly higher than the BSSR of the unaffected contralateral side. The follow-up CT scans revealed a slight remodeling process without relevant osteolysis as previosuly described for similar bony Bankart repairs [15].

The clinical outcome of the patient was excellent as described above. It cannot be ignored that a mere capsulolabral shift without fragment reposition might have resulted in a similar result. However no capsulolabral defect was visible on preoperative MRI scans ot during surgery. Instead the preoperative CT scan analysis revealed an extensive deficiency of the passive stabilization capacity of the bony glenoid. Therefore the primary goal of the surgery was to restore the bony glenoid concavity. Since the possibility of capsular elongation without visible defect exists [16] and due to the fact that the patient is a professional wrestler with high shoulder demand we additionally perfomed a capsuloligamentous shift with the goal to further improve the postoperative stability. The main goal of this case report is, however, not to report the clinical outcome of the procedure which has been presented before [11] but instead to point out the biomechanical effect of glenoid concavity loss and reconstruction even in the absence of glenoid diameter and surface area deficiency.

## Conclusion

This case shows that the passive stabilizing effect of the glenoid can be compromised by loss of concavity despite the absence of loss of articular surface or diameter. Therefore, addressing the concavity loss and resulting reduction of the BSSR is recommended in these cases. Bony Bankart repair was successful in restoring the BSSR of the patients shoulder as determined by mathematical calculations based on CT scans.

## Abbreviations

BSSR: Bony Shoulder Stability Ratio
CT: Computed tomography
WOSI Score: Western Ontario shoulder instability score
ASOSS: Athletic Shoulder Outcome Scoring System (ASOSS)
